# Leg Disorders in Broiler Chickens: Prevalence, Risk Factors and Prevention

**DOI:** 10.1371/journal.pone.0001545

**Published:** 2008-02-06

**Authors:** Toby G. Knowles, Steve C. Kestin, Susan M. Haslam, Steven N. Brown, Laura E. Green, Andrew Butterworth, Stuart J. Pope, Dirk Pfeiffer, Christine J. Nicol

**Affiliations:** 1 School of Veterinary Science, University of Bristol, Langford, Bristol, United Kingdom; 2 Ecology and Epidemiology Group, Biological Sciences, University of Warwick, Coventry, United Kingdom; 3 The Royal Veterinary College, Royal College Street, London, United Kingdom; Katholieke Universiteit Leuven, Belgium

## Abstract

Broiler (meat) chickens have been subjected to intense genetic selection. In the past 50 years, broiler growth rates have increased by over 300% (from 25 g per day to 100 g per day). There is growing societal concern that many broiler chickens have impaired locomotion or are even unable to walk. Here we present the results of a comprehensive survey of commercial flocks which quantifies the risk factors for poor locomotion in broiler chickens. We assessed the walking ability of 51,000 birds, representing 4.8 million birds within 176 flocks. We also obtained information on approximately 150 different management factors associated with each flock. At a mean age of 40 days, over 27.6% of birds in our study showed poor locomotion and 3.3% were almost unable to walk. The high prevalence of poor locomotion occurred despite culling policies designed to remove severely lame birds from flocks. We show that the primary risk factors associated with impaired locomotion and poor leg health are those specifically associated with rate of growth. Factors significantly associated with high gait score included the age of the bird (older birds), visit (second visit to same flock), bird genotype, not feeding whole wheat, a shorter dark period during the day, higher stocking density at the time of assessment, no use of antibiotic, and the use of intact feed pellets. The welfare implications are profound. Worldwide approximately 2×10^10^ broilers are reared within similar husbandry systems. We identify a range of management factors that could be altered to reduce leg health problems, but implementation of these changes would be likely to reduce growth rate and production. A debate on the sustainability of current practice in the production of this important food source is required.

## Introduction

Due to their short reproductive cycle and their worldwide popularity as a food, poultry represent the most highly selected livestock. Selection of broiler chickens (chickens grown for their meat) has been primarily directed at economic traits which have reduced costs of production [1]–[3]. Throughout the world the majority of broilers are reared using very similar, modern, intensive systems of production where birds are confined for their lifetime within high density housing [4] and reared from hatch to slaughter weight within approximately 40 days. However, there is evidence that in optimising traits for production the resulting birds, whilst producing meat at a low cost, have a reduced viability and reduced welfare [5]–[7], with poor walking ability, or locomotion, a primary concern.

Previous research has highlighted associations between management practices and levels of leg disorders. Most attention has focussed on the partially effective practices of reducing feed quantity or the nutrient density of feed [e.g. 8], [9], on providing more than 1-hour of darkness each 24-hour period [10], and on attempts to increase bird activity [11], [12]. There are also known genetic effects, with genotype influences on many traits associated with leg health [7], [13]–[15]. Despite a large body of work investigating the effects of specific risk factors [reviewed in 16], there has been little previous work to examine how these practices interact on real commercial farms to determine the overall level of leg disorders in particular flocks. There are also many management practices, with potential influences on leg disorders, which have not been looked at quantitatively. This study was therefore commissioned by the UK Department for Environment, Food and Rural Affairs to investigate the extent of variation of leg disorders within UK flocks and to identify methods by which these disorders could possibly be controlled.

## Materials and Methods

We studied broiler flocks belonging to five major UK producers who together accounted for over 50 per cent of UK production. Two other relatively large companies were invited to participate but declined. We obtained data from each producer in proportion to their respective annual broiler production. Visits were randomised to farm and flock and were made by veterinarians who had completed a five-day training course to evaluate broiler walking ability with a standardised gait scoring method [7]. Eighteen veterinarians with postgraduate qualifications in poultry medicine and production, or in welfare science, acted as flock assessors and were trained to categorise gait scores within a range from 0 (completely normal) to 5 (unable to stand). The scoring system primarily assesses walking ability rather than exhaustion, with assessors trained to identify rolling gaits, limping, jerky and unsteady movements and problems with manoeuvrability. The scoring system is also known to correlate well with other methods of assessing leg disorders that do not involve active movement, such as the latency-to-lie test [17]. Throughout the study the uniformity of the assessors' scoring was monitored and by the end of the course, average scores for each category were all within half a score. During the subsequent 18 month study, assessors were sent at approximately six and 12 months, a tape containing new video sequences covering a range of gait scores. The scoring of these tapes was monitored to ensure that the assessors remained in agreement. Reference movies of birds' walking ability for each of the six categories are given in the supplementary information [Movie S1, Movie S2, Movie S3, Movie S4, Movie S5, Movie S6]. Each of 176 flocks was visited approximately three days before the flock was depopulated for slaughter and at least 250 birds from each flock were gait-scored from ten, pre-selected, randomised sites within a house.

Fifty seven of the 176 flocks in our study were not ‘depopulated’ for slaughter simultaneously. Instead, one of more groups of birds were removed sequentially over a period of days or weeks in a process known as ‘thinning’. This process involved the removal of a portion of the flock, usually the female birds, to allow the remaining birds more room to grow on to a greater weight. To account for the effects of ‘thinning’ practices, an additional 30 visits were made as second visits approximately three days prior to a later depopulation of one of the original 176 flocks. The flocks visited a second time were also chosen at random from the initial set of flocks.

A primary aim of the study was to investigate possible risk factors associated with the wide inter-flock variation in leg disorders. Of particular interest were risk factors associated with bird husbandry which could possibly be altered when rearing future flocks. Information on these aspects was obtained for each flock by a direct interview with a farm representative. The same questionnaire was used for each visit and comprised 134 questions initially about the breeding flock that had supplied the farm, the facilities where the eggs had been hatched, the distance and time the chicks had been transported, and hatchery vaccination policies. Information was then obtained about the number, weight, sex and time of chicks placed, and their date of arrival. The largest section of the questionnaire sought information on husbandry practices including stocking density and thinning practices, nutritional information, layout and construction of the house, and background information on health, growth rates, mortality and culling policies. Finally, information about the personnel working with the flock, the farm, biosecurity measures and company policies was obtained. After conducting the direct interview, each veterinary assessor collected direct information relating to air quality, temperature, general cleanliness and feed quality.

### Statistical Analysis

Statistical models were built to identify between-flock variables that were associated with the differences in average flock walking ability. The multilevel modelling software package MLwiN v2.01 (http://www.cmm.bristol.ac.uk/MLwiN/index.shtml ) was used as it allowed us to create linear models within the hierarchical structure of the data of repeated measurements on flocks and flocks within companies.

## Results

The overall results of the survey, showing the distribution of gait scores prior to slaughter, are given in Table 1. The figures in Table 1 were calculated using the gait scores of a flock, weighted by the size of the flock as given by the number of birds placed as chicks. The minimum and maximum values in Table 1 show that there was considerable variation in walking ability between flocks, but overall, 27.6 per cent of birds represented by this survey had a gait score of 3 or above.

**Table 1:** The estimated percentage of birds in the survey population within each gait score category.

	Gait Score					
	0	1	2	3	4	5
**Mean**	2.2	26.6	43.5	24.3	3.1	0.2
**SD**	4.8	21.1	15.9	21.3	7.0	0.5
**Min**	0.0	0.0	1.6	0.0	0.0	0.0
**Max**	34.7	82.7	74.6	83.7	45.9	3.2

Mean, SD, minimum and maximum for flocks are shown. The values are calculated from flock averages weighted by birds placed and include first and second visits. Total birds placed n = 4,845,962. Total birds gait scored-206 flocks×minimum of 250 birds per flock-n is approximately 51,000.

Table 2 shows the percentage of birds within each gait score category broken down by the five companies and by the first and second visits. There was a large amount of variation in the distribution of gait score in flocks between the different companies. Company 4 notably produced only 8.5 per cent of birds with gait scores of 3 and above at the first visit compared with 22.7 to 29.7 per cent for the other companies, and only 0.6 per cent of birds with gait scores of 4 and above compared with 1.3 to 4.2 per cent for the other companies. Table 2 also shows the deterioration of gait over time for companies 1, 4 and 5 where a minimum of four second visits were made, the figures for the second visit to company 3 representing only one flock.

**Table 2:** The percentage of birds in each gait score category by producer and by first and second visit.

			Gait Score								
			0	1	2	3	4	5	Mean GS	Birds Placed (n)	Flocks (n)
**First Visit**	**Company**	**1**	2.9	27.0	47.4	20.4	2.1	0.2	1.92	1,484,392	71
		**2**	1.0	21.9	49.1	25.3	2.2	0.5	2.07	191,295	10
		**3**	1.0	21.3	48.0	28.4	1.2	0.1	2.08	486,258	20
		**4**	3.7	44.1	43.6	7.9	0.5	0.1	1.58	773,145	26
		**5**	1.5	29.0	41.2	24.2	3.9	0.3	2.01	1,225,925	49
			**0**	**1**	**2**	**3**	**4**	**5**	**Mean GS**	**Birds Placed (n)**	
**Second Visit**	**Company**	**1**	2.2	8.2	40.3	40.6	8.0	0.7	2.46	206,360	11
		**3**	1.5	53.2	43.1	2.2	0.0	0.0	1.46	34,000	1
		**4**	3.5	26.6	37.5	31.1	1.2	0.1	2.00	119,150	4
		**5**	0.3	4.2	23.7	59.4	11.9	0.4	2.80	329,037	14

The table also shows the mean gait score and the number of birds represented in terms of birds placed.

The average gait score for a flock was modelled in terms of the levels of the risk factors recorded by the veterinary assessors. In addition to the assessment of husbandry variables as linear predictors of average flock gait score, the age at which the birds were assessed, and a seasonal effect, were included in the model. It was necessary to correct for both age and season at the time of assessment, before the effects of different husbandry practices could be properly investigated. The seasonal effect was expected to be cyclical, so was modelled as a sinusoidal curve to identify any cyclical variation in average gait score over a 12 month cycle. Both Sine and Cosine terms for month (January = 1, February = 2, etc) were used in the modelling process allowing us to look for an annual maximum and a minimum effect of season on gait score. A number of variables in the model were centred [Text S1] by subtracting the mean of the variable from each of its measurements.

The most parsimonious multilevel model we obtained of the relationship between the average gait score of a flock, and the risk factors that were recorded within the survey, is shown in Table 3. When company was included as a fixed effect, rather than a random effect, in the model the parameter estimates shown in Table 3 were not meaningfully altered and there were no significant variable/company interactions. Similarly, when the veterinary assessors were included in the model as fixed effects the parameter estimates were not substantively altered. The parameter estimates in the final model are shown and also details of which of the variables are centred. The mean, minimum and maximum of the predictor variables are shown in Table 4, including all those variables which were centred.

**Table 3:** The parameter estimates, their standard error and significance for the model of average flock gait score.

Variable	Variable Type	Parameter Estimate	SE	P
Constant		2.52	0.158	0.000
Season (Sin)	Continuous	−0.099	0.0408	0.016
Season (Cos)	Continuous	−0.035	0.0442	0.463
Age assessed (day)	Continuous (Centred)	0.048	0.0049	0.000
Second visit	Binary	0.25	0.089	0.005
Breed A (% in flock)	Continuous	−0.0024	0.00108	0.025
Dietary wheat (wk 3) %	Continuous	−0.017	0.0078	0.027
Average dark (hr/day)	Continuous (Centred)	−0.079	0.0283	0.005
Stocking density (kg/m^2^)	Continuous (Centred)	0.013	0.0057	0.024
Antibiotic	Binary	−0.17	0.069	0.011
Dusty/broken feed pellets	Binary	−0.15	0.063	0.017

The parameter estimates give the amount of change in average flock gait score for a unit change in each variable. Note, as explained in the text, factors are not and cannot be arranged in order of importance. Positive parameter estimates mean that an increase in the value of a variable is associated with an increase in flock average gait score and negative parameter estimate, a decrease.

**Table 4:** Mean, minimum and maximum values of the continuous predictor variables in the model of average flock gait score.

Variable	Mean	Min	Max
Age assessed (day)	39.8	28	56
Breed A (% in flock)	85.6	0	100
Dietary wheat (wk 3) %	9.2	0	30
Average dark (hr/day)	2.9	0	8.5
Stocking density (kg/m^2^)	31.3	15.9	44.8

**Figure 1:**
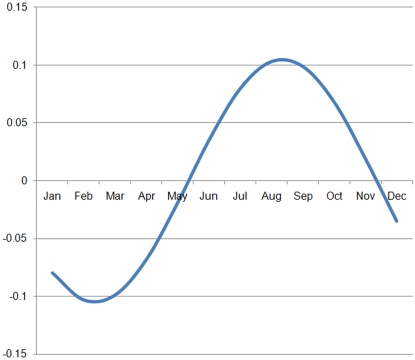
The modelled seasonal change in average flock gait score.

When all other variables were held constant, there was a seasonal pattern to the average gait score of the flocks (Table 3 and Figure 1) with the lowest (best) gait scores occurring in March and the highest (worst) in September. The age at which the birds were assessed was important in determining gait score, with every extra day, across the range of 28 to 56 days, leading to an average daily deterioration in score of 0.048. Although each flock was visited close to slaughter when gait is known to be poorest, within the survey as a whole we were able to evaluate the effect of age on locomotion problems throughout the growth period because of the wide range of age at slaughter. A post-thinning visit was associated with an increased average gait score of 0.25 over and above that due to the age at which the birds were assessed, probably due to the effect of the stress of the first thin, and/or the preponderance of larger, faster-growing male birds remaining in the flock after thinning.

We found that a number of fundamental husbandry practices were significantly associated with average flock gait score and these are detailed below.

A major influence was bird genotype. Broilers worldwide are predominantly of two types, from either one of two major international breeder companies, labelled here A and B. Birds from both genotypes are sometimes reared together within one flock. For every percentage increase in Breed A birds in a flock, from between 0 to 100 per cent, there was a 0.0024 improvement in flock gait score.

Whole wheat is sometimes fed to broilers as part of their diet, predominantly to improve digestive function. For every percentage increase in dietary wheat fed, from 0 to 30 per cent, as measured during their third week of life, there was a 0.017 per cent improvement in flock gait score.

Broilers are reared under a wide variety of artificial lighting regimes. For every 1 hour increase in the daily period of darkness, across the range of 0 to 8.5 hours, there was a 0.079 improvement in flock gait score.

There has been debate about the importance of stocking density as an influence on bird welfare and locomotion [18]. Within limits, putting as many birds in a house as possible for each rearing cycle will improve profitability. For every 1 kg/m^2^ increase in stocking density as measured at the time of the flock assessment, across a range from 15.9 to 44.8 kg/m^2^, there was a 0.013 deterioration in flock gait score.

Antibiotics are routinely used during different stages of broiler rearing and their use can be quite difficult to quantify accurately. In our study farmers were simply asked if a flock had received antibiotic. A reply of ‘yes’ in this context meant that a flock had received an extra antibiotic treatment in addition to that which would be part of normal rearing practice. For the flocks for which the farmer had answered ‘yes’ flock gait score was reduced by 0.17.

Broiler feed is pelleted to minimise wastage and to increase the amount of feed that a bird can consume within a given time. In our study, veterinary assessors made a subjective judgement of whether the quality of the pelleting was good or poor (dusty/broken). For flocks with poor pelleting, gait score was on average 0.15 improved.

**Figure 2:**
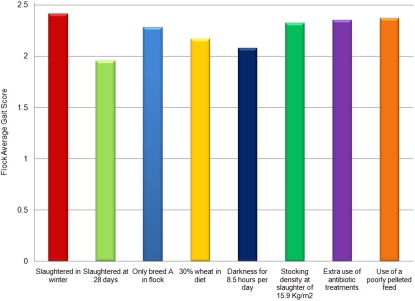
A modelling exercise reveals the extent to which average flock gait score might be improved (i.e. lowered from a notional average score of 2.5) by adoption of the ‘best’ management practice for each variable that we recorded within our study.

The magnitude of a parameter estimate given in Table 3 is not an indicator of the importance of the association of a variable with an increase or a reduction in gait score. This is because the magnitude of the parameter estimate is dependent upon the measurement scale used (e.g. kg or g, hour or day). Further, it is not possible to rank objectively the variables in terms of importance as subjective considerations such as practicality and the perceived cost of altering the husbandry practice have to be considered. For example, the results show that using 100% Breed A birds compared with 100% Breed B birds would reduce average flock gait score by 0.24. To achieve a similar reduction in average gait score by manipulating stocking density alone would require a *reduction* in stocking density of 18.5 kg/m^2^. Despite these considerations there is value in using our model in a predictive capacity to show the improvement in average flock gait score that might be achieved on a hypothetical farm if each relevant variable attained the ‘best’ value actually recorded within the survey. Figure 2 shows the potential improvement from a baseline flock average gait score of 2.5, the middle of the range of possible scores. The Figure shows that no, one variable was associated with a large shift in average flock gait towards the score of 0, a perfect gait, but that the age at which birds are slaughtered appears to be of primary importance, followed by the hours of darkness that the birds are allowed.

## Discussion

For comparison with previous surveys we draw attention to the prevalence of birds in our survey that had gait scores of 3 or above. This cut-off point is important because there is evidence from studies of the effects of analgesic drugs that birds in these categories can be in pain [19]–[22], Other surveys have reported between 14.1% and 30.1% of birds with gait scores of 3 or above in different European countries, although it is not always clear whether weighted or unweighted estimates have been used [7], [23], [24]. Our survey presents a conservative estimate of UK prevalence of leg disorders in meat chickens because the national proportion of birds slaughtered at second, and subsequent thinnings, when gait score tends to deteriorate, is higher than in our sample, and because we present results only from companies that volunteered to participate.

All companies had a policy of culling broilers and some farms separately identified “leg culls” conducted because of leg disorders. If a flock within the survey were to be heavily culled because of leg disorders it would be expected that the overall flock gait score would be improved. However, we found no association between the flock average gait score and the percentage of birds culled as “leg culls”. However, the lack of a relationship may reflect the difficulties farmers have in recording these data in a standardised manner across the survey as a whole. Companies themselves report that these records are inconsistent between farms.

Despite examining a fuller range of husbandry and management practices than previous surveys, we did not identify many novel or previously unreported risks. An effect of season has been noted before in the US, where a higher percentage of leg abnormalities was reported in the summer [25]. The strong genotype effect that we found, confirms the important genetic component to leg disorders, and many of the husbandry effects detected most likely alter levels of leg disorder through direct or indirect effects on growth rate. Thus, we consider the effect of feeding whole wheat is probably due to the slower rate of digestion for whole wheat resulting in reduced growth rate, whilst providing broken or dusty pellets, rather than whole pellets, probably reduced overall consumption rates.

Birds reduce or cease their feed intake during periods of darkness, with associated reductions in growth rate. This is the most likely explanation for the beneficial effect of longer dark periods on the prevalence of leg disorders. The detrimental effect of higher stocking densities may be more complex, reflecting not only a lack of room available for birds to move and exercise, but also the extra environmental loading from increased biomass (e.g. additional ammonia and litter moisture) [18]. Finally, the improvement in leg health observed when antibiotics were used, was probably due to a reduction in the infectious disorders which can cause some types of leg problem.

The study indicates that modern husbandry and genotypes, biased towards economics of production, have been detrimental to poultry welfare in compromising the ability of chickens to walk. However, we demonstrate that within the current framework there is variation in the magnitude of the problem between different flocks, and so some scope to improve walking ability through alterations in husbandry practice. Work needs to be carried out on the predictability of these risks, and the economics of improved welfare practices, for them to gain industry acceptance. An informed balance could then be drawn between profitability and our moral obligation to maintain good standards of animal welfare. The agreement, in May 2007, within the EU of new regulations governing the conditions under which broilers may be reared [26] is a recognition of the problems associated with modern broiler production and is an attempt at a first step towards remedying the situation. The new measures will include the introduction of a maximum stocking density limit, data collection and scientific monitoring of impacts on chicken welfare. The new Directive will not come into force until 2010 but it will prevent farms from stocking birds at densities over 39 kg/m^2^ in subsequent flocks where mortality levels in the past seven flocks have exceeded a set level. The animal welfare implications of monitoring mortality and culling rates are potentially complex. In the short-term, the new Directive could lead to less rigorous culling of birds with leg problems and thereby increase suffering. However, in the longer term, the Directive could act as a stimulus to breeding companies to produce more robust genotypes, with a reduced susceptibility to leg disorders.

Research shows that consumers currently know little about how broiler chickens are reared but can be shocked when presented with information about current commercial practices [27]. Since the sustainability of intensive broiler production depends on continued consumer acceptance of the farming practices involved, the broiler industry will need to work with the scientific community to develop more robust and healthier genotypes and to ensure that optimal husbandry and management practices are fully implemented.

## Supporting Information

Text S1 Further explanation of the linear model of mean-flock-gait-score.
(0.03 MB DOC)

Movie S1 Example of a gait score of 0. Movies S1 to S6 Examples of the gait scores taken from the veterinary assessors' training videos.
(2.19 MB MOV)

Movie S2 Example of a gait score of 1.
(4.23 MB MOV)

Movie S3 Example of a gait score of 2.
(2.67 MB MOV)

Movie S4 Example of a gait score of 3.
(3.02 MB MOV)

Movie S5 Example of a gait score of 4.
(4.25 MB MOV)

Movie S6 Example of a gait score of 5.
(5.71 MB MOV)
