# Supplementary material for: Leg Disorders in Broiler Chickens: Prevalence, Risk Factors and Prevention
Source: PLoS One. 2008 Feb 6;3(2):e1545. doi: 10.1371/journal.pone.0001545 (PMC2212134; doi:10.1371/journal.pone.0001545)
Supplement: Text S1 — Further explanation of the linear model of mean-flock-gait-score. (0.03 MB DOC) [file pone.0001545.s001.doc]

Centring a predictor variable is a common practice in this type of mathematical modelling. It aids interpretation of both the constant in the model, and of parameter estimates for the centred predictor variables, as the parameter estimate then show how the response variable changes for a given off-set from the mean of that predictor variable. For example, in a simple model where the response variable mean-flock-gait-score is predicted only by stocking density, if stocking density were not centred, the constant in the model would be interpreted as the mean gait score when stocking density was equal to 0. In a model in which stocking density were centred, the constant would represent the mean-flock-gait-score at the mean stocking density, a far more meaningful and comprehensible representation.

The constant term in a linear model ties the model to a particular starting value. It is useful if the constant is interpretable. In our model the constant of 2.52 gives the average flock gait score for a flock visited at the average age of 39.8 days, on the average dark period of 2.9 hrs/day, at the average stocking density of 31.3 kg/m^2^, at the first visit/thinning, with no Breed A birds, no wheat in the diet, without antibiotic, on feed of good physical quality and slaughtered in the month of May or November.

Within our model 52 per cent of the variance in flock gait score was explained. This is an estimate adjusted for the number of predictor variables in the model and is a relatively high percentage for this type of study.
